# Obsessive compulsive disorder: a case of extreme obsessional slowness in an 18-year-old presenting to the national OCD unit

**DOI:** 10.1192/bjo.2021.337

**Published:** 2021-06-18

**Authors:** Claire Fischer, Ilenia Pampaloni, Sarah Gardiner

**Affiliations:** South West London and St George's Mental Health Trust

## Abstract

**Objective:**

Obsessional slowness in OCD is a rare phenomenon on which there is minimal published literature. This is a particularly severe and atypical case of early onset OCD with extreme obsessional slowness and mutism. To the best of our knowledge, there have been no reports of similar severity published in this age group. This report seeks to provide discussion of important organic causes that may need to be considered as well as information on treatment approach.

**Case report:**

An 18-year-old male was admitted to the National OCD Unit, Springfield Hospital with a history of autism and normal development until the age of 14, after which symptoms of OCD with fear of contamination emerged, followed by progressive motor slowness and mutism.

Due to the severity of OCD and self-neglect he had two previous admissions to CAMHS wards and required a course of ECT to treat catatonic symptoms age 17.

Pharmacological treatment has included Aripiprazole 5 mg and Fluoxetine 60 mg, which the patient was taking at admission. The latter was subsequently switched to Sertraline 250 mg and Aripiprazole increased. As it was hypothesized that his obsessional slowness stemmed from severe levels of anxiety, Buspirone was also added.

Therapy has been intensive, although communication difficulties have made targeting specific fears challenging as the exact nature of the intrusive thoughts remains unclear.

**Discussion:**

Following combined neurology and neuropsychiatry review, the patient spent four weeks in a general hospital for further investigation as it was initially felt an organic cause was likely. Initial differentials included Juvenile Onset Parkinson's or Wilson's disease. Both were subsequently ruled out and despite multiple investigations, no obvious organic cause was found. A markedly abnormal FDG PET scan showed findings usually seen in advanced dementia, but not necessarily clinically correlating to his current presentation.

The OCD unit have continued to provide intensive input and tailored treatment programme, encouraging actions against any rules he has in place. Prompting and pacing, verbal exercises and regular stretching exercises due to stooped posture which he attributed to needing to obey certain rules have been used.

**Conclusion:**

It is important for clinicians to be aware of obsessional slowness in OCD and this report highlights a particularly rare and severe example in a young adult who has been difficult to treat. Organic causes may need to be considered and MDT approach to treatment is essential.

